# “Happiness in the air?” the effects of air pollution on adolescent happiness

**DOI:** 10.1186/s12889-019-7119-0

**Published:** 2019-06-21

**Authors:** Wen-Hsu Lin, Wen-Chi Pan, Chin-Chun Yi

**Affiliations:** 1 Institute of Health and Welfare Policy, College of Medicine, National Yang-Ming University, No.155, Sec.2, Linong Street, Taipei, 112 Taiwan; 2 Institute of Environmental and Occupational Health Sciences, College of Medicine, National Yang-Ming University, No.155, Sec.2, Linong Street, Taipei, 112 Taiwan; 3 Institute of Sociology, Academia Sinica, 128 Sec. 2 Academia Rd., Nankang, Taipei, 11529 Taiwan

**Keywords:** Air pollution, Positive affect, Happiness, Adolescent, Multilevel analysis

## Abstract

**Background:**

We aimed to examine the effect of ambient air pollution at the district level on adolescents’ happiness and their change in happiness over time in a cohort sample from Taiwan.

**Method:**

A cohort from the Taiwan Youth Project was evaluated. The adolescents (*n* = 2571) were in the 7th grade (mean age = 14.3 years) when the study was initiated and resided in 40 districts in three cities and counties in northern Taiwan. We examined the effects of the concentration level of air pollution, including PM_2.5_, PM_10_, and NO_2_, at the district level on adolescents’ happiness and their change in happiness over time (7th to 9th grade). Due to the high correlations of the three pollutants, we examined each separately with similar covariates. The analyses were based on both multilevel modeling and latent growth curve modeling.

**Results:**

Higher concentration levels of each of the three air pollutants measured were associated with adolescent happiness such that a higher level of concentration was related to lower levels of adolescents’ happiness. These results were observed after controlling for important individual- and district-level covariates. However, further analyses did not reveal that the concentration level of air pollution was associated with the change in happiness in the study period (after 3 years). Some sensitivity checks (e.g., adjusting district size) did not change the substantive results.

**Conclusion:**

Many previous studies have shown the influence of air pollution on physical health and negative emotions, but only a few using adult samples have shown that air pollution is inversely related to positive wellbeing. This study may be the first to examine the effects of air pollution on adolescents’ positive affect. Our results echo recent research on the consequent health burden of air pollution. Given that positive affect has been linked to future adult health, the results of the current study provide empirical grounds for early intervention concerning environmental factors.

**Electronic supplementary material:**

The online version of this article (10.1186/s12889-019-7119-0) contains supplementary material, which is available to authorized users.

## Background

Recent developments in positive psychology have initiated great efforts toward researching the impact of positive wellbeing on health, and the results from previous studies have demonstrated that positive wellbeing can act in various ways to enhance health: by directly influencing the physiological system, such as increasing immune competence or reducing prolonged activation of various systems (e.g., neuroendocrine or cardiovascular systems) [1]; by buffering the negative effects of stress on an individual; by building healthy habits; or by reducing risky behaviors [2].

Consistent with the development of positive psychology, the important topic of positive youth development has also gained great attention [3]. The life course theory argues that life stages are linked [4]; that is, an incident in one life stage influences not only that particular stage but also later life stages. Based on this view, a healthy adulthood may be due in part to a well-managed adolescence. In line with this, some scholars have argued that one way to enjoy health in later life is to promote positive wellbeing (e.g., positive emotions, self-esteem, and a sense of acceptance) and positive affect during adolescence [2, 5]. In fact, studies show that positive emotion (e.g., happiness) and wellbeing during adolescence are beneficial for young adults in that they promote subjective health and reduce behaviors that compromise health [5]. Furthermore, positive affect and wellbeing in adolescence have also been related to a lower risk of illness and mortality in later life [6, 7]. Steptoe et al. [1] argued that positive affect may have distinct beneficial effects on an individual independent of negative affect (e.g., depression) [8]. Given the independent benefits of positive affect, it is crucial to gain further insight into the factors that can promote or compromise it.

Previous studies have indicated factors (e.g., school, friends and parent-child relationship) that either promote or disturb positive affect [9, 10], but one possible important factor that has been ignored is air pollution, which was named by the World Health Organization (WHO) as one of the greatest threats to human health in modern society [11]. Some scholars have mentioned air pollution as an important, modifiable, and global public health threat [12]. Although the negative effects of air pollution on physical health, such as respiratory and cardiovascular diseases [13], have been well documented, scholars have also demonstrated that air pollution negatively affects individuals’ mental health and emotions [14, 15]. Based on results from laboratory and morphological studies, scholars have shown that air pollution (e.g., particle matters and nitrogen dioxide) is related to oxidative stress and inflammation in the brain, which in turn is associated with negative effects and related behaviors [13, 16]. In contrast, only a few studies have investigated the possible effects of air pollution on positive developments (e.g., positive affect or wellbeing). Results from these few studies have revealed that air pollution is negatively related to subjective wellbeing [17, 18], life satisfaction [19], and happiness [20]. Especially important was Li et al.’s recent review [21], which reasoned that air pollution can be seen as a stressor and can compromise one’s positive affect via cognitive appraisal or negative feelings (e.g., disgust). This conclusion provided a theoretical basis to connect air pollution to individual positive affect and wellbeing.

While there have been some research endeavors in this area, four limitations can be further improved upon. First, many previous studies have focused on adult or mixed samples [20, 22], but not on adolescents. Given that adolescence is an important life stage with many transitions (e.g., elementary to middle school) and brain maturation, although slower than that in childhood [23], it is crucial to understand how air pollution influences adolescent positive affect. Some scholars have even argued that the concern of public health ought to be on the effects of air pollution on the brain development of children and adolescents [16]. Second, many previous studies did not consider one important air pollutant: PM_2.5_ (particulate matter with aerodynamic diameter ≤ 2.5 μ). The WHO has regularly monitored PM_2.5_ and considered it as one of several pollutants related to human health [11]. Third, many previous studies have presented the relationship of air pollution with positive affect and wellbeing at the country level [24, 25], and although this provides invaluable cross-cultural comparisons, the results may be difficult to infer at the individual level (e.g., ecological fallacy). Finally, in our awareness, no study has investigated how air pollution influences the change in positive emotion during early adolescence. One recent study demonstrated that the variation in green spaces around one’s living area is correlated with one’s mental health [26]. Furthermore, this protective effect varied by age, with older people receiving more benefits than the younger ones (i.e., the protective effect is significant and stronger at a later age) [26]. The extent to which this may also be applied to adolescents’ positive wellbeing and air pollution remains an important empirical question. Consequently, the current literature seems to lack studies on how air pollution influences adolescents’ positive emotion in a non-Western society and the change in their positive emotion during early adolescence. This study tried to fill these gaps by merging data from a longitudinal cohort sample with that from the Environmental Protection Administration (EPA), Taiwan. Hence, it provides a great opportunity to evaluate how living in areas with three specific air pollutants influences adolescents’ (7th–9th graders’) happiness. The research objectives were twofold; this was an attempt to investigate for the first time (1) if adolescents who live in a district that has a higher level of concentration of air pollution have a lower level of happiness than adolescents from districts with a lower concentration; (2) if air pollution influences the change in adolescents’ happiness during early adolescence.

## Method

The data for the present study were drawn from the first three waves of J1 cohorts of the Taiwan Youth Project (TYP), which was conducted by the Institute of Sociology, Academia Sinica, Taiwan. The TYP was a nine-year longitudinal research project that began in 2000. The participants were followed annually for nine years, and the study included two cohorts: (a) J1 (7th graders with an average age of 13 years) and (b) J3 cohort (9th graders with an average age of 15 years). The participants in these two cohorts were selected based on stratified cluster sampling. The research team selected two counties (Taipei and Yilan) and one city (Taipei) in northern Taiwan, which were then stratified further based on different levels of urbanization. After the strata were determined, the cluster sampling method was employed to select the participants. On the survey date, the trained assistants explained to students about the research and students were free to choose whether they wanted to participate or not. Each student who agreed to participate completed a self-administered questionnaire during regular class hours. The first wave (baseline) of data collection occurred in March 2000, and all adolescents (mean age = 13.4 years) from the selected classes participated (*n* = 2690). These students were targeted approximately annually during the junior high school period (grades 7th to 9th). The final analytic sample was based on students who participated in all three waves, provided important demographic information at baseline, and resided in approximately 40 different districts as determined based on their zip code.

### Measures

*Happiness (waves 1, 2, and 3)*. We used the students’ self-reported subjective happiness as the proxy measure of positive affect. While this may appear somewhat simplistic, this particular item has been recognized as important when studying wellbeing [27] and has been used to captured this positive emotion in previous studies [19, 28, 29]. The item specifically asked students, “In general, are you happy lately?” (1 = very unhappy; 2 = unhappy; 3 = happy; and 4 = very happy). The same item was used for all three waves. This item was revers coded for later analyses.

*Air pollution*. In the present study we included two air pollutants: particulate matter, including both ambient PM_10_ (aerodynamic diameter ≤ 10 μ) and PM_2.5_ (aerodynamic diameter ≤ 2.5 μ), and nitrogen dioxide (NO_2_). The original data for the three pollutants were collected from the 17 air quality monitoring stations in Taipei Metropolitan and two air quality monitoring stations in Yilan County. The Environmental Protection Administration (EPA), Taiwan, provided the data for both New Taipei City and Taipei City for Yilan county. However, students came from various districts of Taipei City, New Taipei City, and Yilan County. In order to properly estimate concentration levels of the three pollutants in each district, land use regression (LUR) for PM_2.5_ was adopted because LUR-based estimations usually outperform Kriging-based models in terms of PM_2.5_ prediction (adjusted R-squared = 0.89) [30]. The LUR model used in this study was developed by a research team that considered not only meteorological factors but also cultural ones (e.g., incense from temples) to estimate the spatial-temporal variability of PM_2.5_ from the EPA to each district [30]. Specifically, this LUR model incorporated five GIS datasets: the national land use inventory, map of the industrial park, landmark database, digital road network map, and Digital Terrain Model. Due to data limitations, only the Digital Terrain Model had a specific resolution (40 m × 40 m). In addition, this LUR model also employed Normalized Difference Vegetation Index (250 m × 250 m resolution) images with buffering analyses so that the spatial-temporal variation in the greenness around the monitor stations could be incorporated. This model provided a high explanatory power for estimating PM_2.5_ (e.g., adjusted R^2^ = .89), although only with 17 observation sites. Consequently, while this model may not be perfect because of the limited number of observation sites, it included various important datasets as well as greenness and culturally specific pollution sites (e.g., Taoism tempo), which provides sufficient estimation. However, PM_2.5_ data were not available for the first two waves of TYP (i.e., 2000–2001), which was our exposure time period. Therefore, we used the estimated PM_10_ from the ordinary Kriging method to obtain the concentration level of PM_2.5_ for each district. In other words, from the 2006 data, for the first year when data on PM_2.5_ are available, we calculated the ratio of PM_2.5_ and PM_10_ pollutants and then employed this ratio to estimate the concentration level of PM_2.5_ for each district during the 2000–2001 period. A similar calculation method was used in a previous study [31].

With regard to PM_10_ and NO_2_, the ordinary Kriging method was employed. The reason for not using similar LUR-based estimation is that a satisfactory LUR model for these gaseous pollutants (e.g., NO_2_) has not been developed yet (i.e., a model that takes into account the local specific pollutant source). As such, this study employed a method different from that of PM_2.5_ so that a satisfactory estimation could be reached.

In this study, the exposure to the pollutants was calculated as the average concentration level from 2000 to the month in which the second survey was administered (i.e., March 2001). In other words, we calculate the average concentration level across these months (i.e., January 2000 to March 2001) for each district. Although this exposure period may be somewhat arbitrary, two previous studies showed that air pollution exerted the strongest negative effects at approximately 12 months of exposure time [14, 31]. Although the effect described was on negative emotion, with no clear guide for the effects of air pollution on positive wellbeing, we propose that the designated exposure period is reasonable. To further ensure that all the participants were exposed to the air pollution as calculated, we checked adolescent self-reports on the duration for which they had been residing at the address specified. We excluded those who reported less than a year (less than 1%) of residence. Consequently, all the adolescents in the current study were exposed to the estimated air pollution during the exposure period evaluated.

#### Individual level covariates (waves 1)

In this study, several important individual covariates were included: sex, family income, family intactness, class rank, negative life event, low family cohesion, self-esteem, negative emotion, deviance, and low friends’ support. Many of these variables have been found to be related to individual happiness [32–34]. *Sex* was a dichotomized variable with female as the reference group (49%); *family monthly income* was based on parental report, but when parents did not report, the students’ reports were used; *family intactness* (both parents are at home regardless if they are biological parents) was a dichotomized variable with intact family as the reference group (88%); *class rank* was measured using students’ self-report on their class rank in the last semester and included five categories with a higher score indicating a lower class rank (i.e., low school performance); *negative life event* was the summation of 20 major negative life events that may have happened in the adolescents’ lives with a high score indicating an experience of more negative life events; *low family cohesion* was measured by taking the mean of six items (all on a four-point scale) to capture emotional bonding and adaptability in the family with a higher score indicating a lower level of family cohesion; *self-esteem* was the summation of six items (all on a four-point scale) with a high score indicating a lower level of self-esteem; *negative emotion* was the summation of three self-reported negative emotions (e.g., depression, loneliness, and worrisome) with a higher score indicating a higher level of negative emotion; *deviance* was the summation of the count of self-reported deviant behaviors (e.g., smoking, alcohol use, and skipping class); and *low friends’ support* was measured by taking the mean of three self-report items (e.g., my friends care about me) each rated on a four-point scale. The detailed survey items used in this study, which were developed by the TYP research team, were available in the Table S1 in the Additional file 1.

#### District-level covariates

In the current study, we also incorporated three district-level covariates: the *proportion of district college graduates, population density* and *mutuality*. The former two variables were derived from the open public data from the Ministry of Internal Affairs. *The proportion of college graduates* was used to attempt to capture district socioeconomic status, as previous studies had shown that neighborhood socioeconomic status is related to residents’ mental health [35]. *Population density* of a district was calculated by dividing year-end population of a district by the district size (km^2^), which was found to be related to residents’ wellbeing [36]. *Mutuality*, which captured social cohesion, was an aggregated variable derived from one self-report items regarding subjective feelings of reciprocity among residences in the same area (e.g., “Do you think people in your neighborhood are willing to help others or do they only care about themselves?”). This item was used by previous study to capture community effects on adolescent wellbeing [37].

### Data analysis

**Fig. 1:**
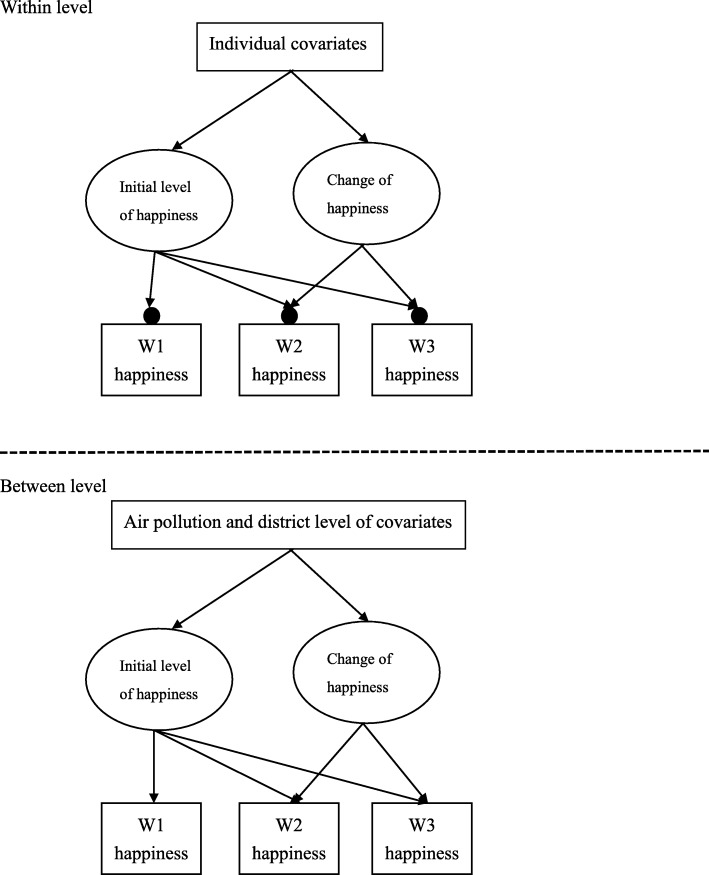
Illustration of three level model in the present study.*Each filled circle at the end of the arrows at the with-level indicates random intercept, which varies across district.

This study tried to first examine whether the three air pollutants at the district level were related to adolescent happiness at wave 2 because of air pollution data in hand. Then, this study further analyzed if the change in happiness during early adolescence also was related to air pollution. For the first analysis, the data structure included analytical levels based on adolescents in different districts. Hence, we employed a hierarchical linear model. Specifically, the present study estimated results using the combined equation below:

$$ {y}_{ij}=\left({\gamma}_{00}+{\gamma}_{01}\left( proportion\ of\ college\ graduates\right)+{\gamma}_{02}\left( Population\ density\right)+{\gamma}_{03}(Mutuality)+{\gamma}_{04}\left({PM}_{2.5}\right)\right)+{\beta}_{1-10}{x}_{ij}+\left({\varepsilon}_{ij}+{u}_{0j}\right) $$

where *y*_*ij*_ is i^th^ adolescents’ happiness in j^th^ district, *γ*_00_ is the overall intercept across all districts, *γ*_*s*_ are the effects of district on individual happiness with *γ*_04_ as the focal point (i.e., the air pollution effect), *β*_1 − 11_ captured the ten individual level covariate effects (e.g., sex, family income, and negative emotion), and *ε*_*ij*_ + *u*_0*j*_ was level 1 residual and level 2 randomness (i.e., deviation from the overall intercept). Given that the focus was on the effects of air pollution on adolescent happiness, a random slope model was not included. In addition, the sources of most forms of air pollution are similar; consequently, there exists a high correlation between different air pollutants [13]. Therefore, the analyses were conducted for each pollutant separately. For the second analysis, the data structure had three levels: time, adolescent, and district. Consequently, we fitted a latent growth curve model of happiness into the district data. In this analysis, the model tried to estimate possible effects of the third-level variables (i.e., the three air pollutants) on the two major growth factors: intercept (i.e., initial level of happiness at wave 1) and slope (i.e., changing rate of happiness) (see Fig. 1 for the model). Furthermore, to make all interpretations meaningful, all the variables were grand mean centered in the subsequent analyses. The present study used full information maximum likelihood (FIML) to account for missing data [38]. All analyses were conducted by using Mplus7.1 [39].

## Results

Table 1 shows that the three air pollutants varied greatly: 34.6 to 72.26 for PM_10_; 20.83 to 35.81 for PM_2.5_; and 20.43 to 34.01 for NO_2_. One interesting point was that the maximum concentration level of PM_2.5_ at 35.81 reached the orange alarm, which is “unhealthy for sensitive groups” according to the Air Quality Index (AQI) of the EPA, Taiwan. The levels of concentration for each pollutant were higher than those in Western countries (e.g., the US and EU countries) [40] but similar to neighboring countries, such as that in Korea [14]. Similarly, the socioeconomic status of each district also varied greatly, with 2.05 to 35.5% of residents graduating from college. The average happiness for these adolescents was reported as being at the “feel happy” level (2.08).

**Table 1:** Descriptive statistics for all variables^12^

	Minimum	Maximum	Mean	SD
PM_2.5_	20.83	35.81	28.11	4.78
PM_10_	34.60	72.26	51.47	9.57
NO_2_	20.43	34.01	28.51	4.47
% of college graduates	2.05	35.54	11.21	8.28
Population density	51	39,947	9310.9	10,543.03
Mutuality	1	2.25	1.70	.200
Wave 1 Happiness	1	4	2.97	.806
Wave 2 Happiness	1	4	2.92	.798
Wave 3 Happiness	1	4	2.83	.763
Sex	0	1	.51	.50
Family income	0	600	54.66	43.60
Family intactness	0	1	.12	.33
Wave 1 Class rank	1	5	2.95	1.18
Wave 1 Negative life event	0	16	2.48	2.10
Wave 1 Family cohesion	1	4	1.99	.64
Wave 1 Self-esteem	6	24	16.02	2.89
Wave 1 Negative emotion	0	4	.57	.80
Wave 1 deviance	0	10	.49	1.01
Wave 1 Friends’ support	1	4	1.76	.54

^1^ *n* = 40 at the district level

^2^ n varied from 2548 to 2571 at the individual level

Table 2 outlines the main focus of our research. In the first column of Table 2, one can see that the effect of PM_2.5_ was positive and significant (β = .01). In other words, the higher the concentration level of PM_2.5_ in a district, the more likely youths from that same district claimed to feel unhappy. This finding is important because the same model controlled for various important covariates, and many of these variables also showed significant effects. For example, as expected, the levels of negative emotion (β = .10), self-esteem (β = .03), number of life events (β = .02), and family cohesion (β = .16) were all significantly related to happiness. Similarly, the second and third columns revealed similar results: both PM_10_ (β = .004) and NO_2_ (β = .01) were positively related to adolescent happiness after controlling for individual- and district-level confounders. Taken together, adolescents from more polluted districts (e.g., those with higher levels of PM_2.5_ concentrations) were more likely to have lower levels of positive affect.

**Table 2:** Multilevel regression analyses^1^

	Model1	Model2	Model3
Intercept	2.92(.02)**	2.92(.02)**	2.92(.02)**
PM_2.5_	−.02(.006)*		
PM_10_		−.01(.003)*	
NO_2_			−.013(.006)*
% of college graduates	−.002(.003)	−.003(.003)	.001(.003)
Population density^a^	–	–	–
Mutuality	−.17(.13)	−.24(.15)†	−.11(.12)
Male	.05(.04)	−.04(.04)	−.04(.04)
Family income^a^	–	–	–
Non-intact family	−.09(.05)†	−.09(.05)†	−.09(.05)†
Wave 1 Class rank	.009(.013)	.008(.01)	.009(.013)
Wave 1 Negative life event	−.02(.01)**	−.02(.01)**	−.02(.01)**
Wave 1 Low family cohesion	−.08(.03)**	−.08(.03)**	−.08(.03)**
Wave 1 Self-esteem	.03(.004)**	.03(.004)**	.03(.004)**
Wave 1 Negative emotion	−.10(.03)**	−.10(.03)**	−.10(.03)**
Wave 1 deviance	−.003(.02)	−.003(.02)	−.003(.02)
Wave 1 Low Friends’ support	−.11(.04)**	−.11(.04)**	−.11(.04)**
Wave 1 Happiness	.18(.03)**	.18(.03)**	.18(.03)**

*n*_1_ = 2538; n_2_ = 40.

†*p* < .1;*p < .05; **p < .01.

^1^The presented number is the estimated coefficient and standard error is in the parentheses

^a^ The effects of these two variables were estimated but very small because of original measurement scale. Hence, they were not presented here

Table 3 presents the three-level results (Model 1 to Model 3). All the models acceptably fit the data based on three fit indices (χ^2^, CFI (comparative fit index) and RMSEA (root mean square error of approximation)). The basic growth curve model (note3 of Table 3 at the bottom) as well as the three level analyses demonstrated that the change in adolescents’ happiness was significant with negative sign, which indicated that adolescents’ happiness during these three years gradually decreased. Results of Models 1–3 of Table 3 were fairly similar to those shown in Table 2. The three air pollutants showed significant effects on the initial level of happiness (β = −.01) for all three pollutants. Because the initial level was set at wave 1, these results also implicitly pointed out that long-term air pollution exposure influenced adolescents’ happiness in grade 7. When combined with Table 2 results, this also means that long-term exposure to the three air pollutants affects adolescents’ happiness in both grades. However, the three pollutants did not have significant effects on the change in happiness over the three-year period. Similarly, most of our important district controls did not have significant relationships with either the initial level or the change of happiness. In contrast, some of the individual variables had different associations with the change in happiness in adolescence. For example, low family cohesion not only influenced adolescents’ happiness in 7th grade (β = −.25) but also “accelerated” the decline of their happiness (β = .09). Similar effects were found for self-esteem, negative emotions, and low Friends’ social support.

**Table 3:** Multilevel latent growth curve analyses^123^

	Model 1		Model 2		Model 3	
	Initial level of happiness	Change of happiness	Initial level of happiness	Change of happiness	Initial level of happiness	Change of happiness
PM_2.5_	−.01(.006)*	−.003(.004)				
PM_10_			−.01(.003)**	−.001(.002)		
NO_2_					−.01(.01)*	−.003(.004)
% of college graduates	.001(.002)	−.001(.001)	−.001(.002)	−.001(.001)	.002(.002)	−.001(.001)
Population density^a^	–	–	-†	–	–	–
Mutuality^b^	−.09(.10)	–	−.19(.11)†	–	−.03(.10)	–
Male	.02(.03)	.04(.02)*	.02(.03)	−.04(.02)*	.02(.03)	−.04(.02)*
Family income^a^	–	–	–		–	
Non-intact family^b^	−.06(.04)	–	−.06(.04)	–	−.06(.04)	–
Wave 1 Class rank	.02(.01)	.02(.01)	.02(.01)	.02(.01)	.02(.01)	.02(.01)
Wave 1 Negative life event	−.02(.008)*	.002(.004)	−.02(.008)*	.002(.004)	−.02(.008)*	.002(.004)
Wave 1 Low family cohesion	−.25(.03)**	.09(.02)**	−.25(.03)**	.09(.02)**	−.25(.03)**	.09(.02)**
Wave 1 Self-esteem	.04(.004)**	−.01(.003)**	.04(.004)**	−.01(.003)**	.04(.004)**	−.01(.003)**
Wave 1 Negative emotion	−.25(.02)**	.07(.01)**	−.25(.02)**	.07(.01)**	−.25(.02)**	.07(.01)**
Wave 1 Low peer support	−.17(.03)**	.03(.02)†	−.17(.03)**	.03(.02)†	−.17(.03)**	.03(.02)†
Wave 1 deviance	.01(.01)	−.001(.009)	.01(.01)	−.001(.009)	.01(.01)	−.001(.009)

n_1_ = 2538; n_2_ = 40

†*p* < .1;*p < .05; **p < .01

^1^The presented number is the estimated coefficient and standard error is in the parentheses

^2^Model fit for each of the four models is acceptable, which may be obtained from the first author upon the request

^3^The basic growth curve model had marginal fit (χ^2^ (1) = 13; CFI = .98; RMSEA = .068) with both initial level and change of happiness are all significant (initial level = 2.98**; change = −.10**).When estimated in three, both initial level and change of happiness remained significant with identical signs (initial level = 2.97**; change = −.067**)

^a^The effects of these two variables were estimated but very small because of original measurement scale. Hence, they were not presented here

^b^The effects of these two variables on the change of happiness are not estimated because these effects caused model estimation problems due to very small effect

While the average district sample size was approximately 61, some districts had as few as three adolescents (e.g., (see Table 4 in Appendix)). We reran the models and excluded districts with fewer than 10 youths, which brought the level 2 sample size to 35. The results were fairly similar. For example, the effect of PM_2.5_ on happiness remained significant and similar in size (β = −.02). Consequently, even with the risk of low power (i.e., losing level 2 units), the results remained significant. Similarly, some districts may be bigger than others (e.g., size of the district in km^2^); hence, the concentration level in each residential area may vary greatly. We reran all our analyses by excluding districts that are greater than 85 km^2^ (i.e., 1 SD above the mean). Thus, the analyses were based on 34 districts. The results remained similar regarding the effects of pollutants on adolescent happiness. However, the influence of NO_2_ became marginally significant (β = −.01; *p* = .055). Finally, it may also be argued that individual happiness may be influenced by both the concentration of a particular air pollutant in that district and the neighboring districts’ spatial autocorrelation. To evaluate the likelihood of this possibility, we first calculated the two global Moran’s Is with different weight matrixes (queen and rook matrix) for each pollutant. If each of the global Moran’s I is not significant, the spatial correlation may be at a minimum. Across all three air pollutants, all tests (adjust for α) showed non-significant results. As such, we did not pursue this route further (results can be obtained upon request). Similarly, the first two sensitivity analyses were repeated for the three-level model and the results were all substantively similar to the results described above. However, one caution for these sensitivity analyses for the three-level model was the instability of the model (i.e., some estimated coefficient became very small) because of low district sample size.

## Discussion

While research-based attention to positive health has been growing, much remains unknown about the factors related to adolescents’ positive affect. Although previous studies have shown some factors related to adolescents’ positive affect, such as family structure [41], recent environmental research demonstrated that ambient air pollution might also be a potential factor influencing wellbeing and positive affect [7, 17, 20].

While many previous studies conducted investigations at the country level or used adult samples from Western countries, which often have a relatively low level of air pollution [40], to the best of our knowledge, this is the first study to consider the association between adolescent happiness, its change, and long-term exposure to air pollution in a non-Western society. A particularly important fact is that this study considered three air pollutants, among which PM_2.5_ is the most important. The results from multilevel analyses showed that during early adolescence (7th to 9th grade), the concentration levels of PM_2.5_, PM_10_, and NO_2_ were related to adolescent happiness such that a higher level of air pollution was related to a lower level of happiness. These results were similar to two previous studies, though both studies differed greatly from the current study. One study showed that several pollutants were related to a lower level of happiness [19], but it did not focus on adolescents. The other [20] showed that perceived air pollution (e.g., perceived hazard of air pollution) was related to a lower level of happiness. Together, these studies concluded that air pollution was negatively correlated with individuals’ positive affect, and this is in addition to the negative effects already mentioned, including depression [11] and deteriorated physical health [14].

This study went further to examine the relationship between long-term air pollution exposure and the change in happiness. However, the results did not show significant effects of these three air pollutants on change in adolescents’ happiness during the three years of junior high school. Although one previous study [26] revealed that green spaces may have protective benefits for adults, such effects did not present at the general level but in a specific age period (e.g., among women in late adulthood). Furthermore, in Astell-Burt et al.’s [26] study, green space was a time-varying variable that provided information on a specific effect (i.e., the effect at a particular age) [42]. In contrast, our analyses focused on long-term air pollution exposure as the time-independent variable as it related to the “trajectory” of happiness in adolescence. Consequently, these results may not be so different from the previous study. In fact, our results from both analyses implied that air pollution is significantly related to adolescent happiness at both wave 1 (i.e., the initial level in Table 1) and wave 2 (i.e., the results in Table 2). We conducted similar additional analyses with wave 3 happiness and the results were substantively similar to those shown in Table 2. Hence, we tentatively showed that at each time point, long-term exposure was related to a lower level of happiness. Therefore, combining the results from this and the previous study [26], long-term air pollution or green space exposure might be only weakly related to the general change in an individual’s mental health but might significantly influence mental health at a specific age or age period.

The relationship revealed between air pollutants and happiness can be viewed in two ways. First, studies have shown air pollutants can penetrate into the lung compartment, thereby entering the systemic circulation and reaching the brain. In turn, they damage or interfere with brain function and the central nerve system by causing inflammation and oxidative stress [43]. This process can influence many regions of the brain, such as the prefrontal cortex [44], which includes the ventromedial prefrontal cortex and anterior cingulate cortex related to happiness [45]. Furthermore, scholars have mentioned that striatum, which includes components related to emotions (e.g., nucleus accumbens), is particularly susceptible to air pollution [46]. One study [12] mentioned that inflammatory reactions in the brain also impair neurotransmitter signaling, which may influence dopamine and serotine regulation, thus influencing emotions (e.g., happiness). Second, exposure to air pollutants, particularly PM_2.5_, may exacerbate physical health [47] to cause a low level of wellbeing. This indirect effect is congruent with literature on stress that often considers physical illness as a type of stress [48]. Similar to this indirect association, one study showed that exposure to air pollution (e.g.,PM_2.5_) is also related to high perceived stress, which may also compromise one’s positive wellbeing.

This study has several strengths. First, it employed longitudinal data with more than 2000 adolescents. Second, it is the first to focus on positive emotion in Asian adolescents. Finally, it also included three major air pollutants. Although this study provides the first and invaluable insight into the effect of air pollution on adolescents’ happiness, it also has several limitations. First, the measure of air pollution was based on a specific LUR model. While this model is suitable and useful, the estimation may not be perfect. In addition, the PM_2.5_ level was indirectly estimated through the inverse ratio between PM_2.5_ and PM_10_. Second, this study did not include indoor air quality information, which is related to individual development in addition to outdoor pollution [16]. Third, the sample size of districts was low. However, a recent simulation study suggests that a level 2 sample larger than 30 is needed to have a nearly unbiased estimation of variance component [49]. Consequently, while the present sample size was only moderate, it nevertheless may be sufficient for the present analyses. Fourth, we measured air pollution at the district level, which implicitly assumes that the exposure is the same for all individuals in a particular district. This may not be as great a problem for particulate matter as for NO_2_ because particulate matter is more spatially homogeneous [13] and is a regional pollutant [50]. Finally, a firm causal relationship cannot be established given that many covariates were not included, with the most relevant one being physical illness status, such as asthma [13]. Future studies may further this line of research in several ways. For example, studies may consider not just happiness but also other important positive aspects of emotion/wellbeing, such as life satisfaction and self-esteem. Second, while this study incorporated many important air pollutants, future studies may try to include others such as sulfur dioxide or black carbon. Finally, future studies may also include other measures of environmental risk, such as noise or lack of green spaces.

A recent WHO warning [51] on the effects of air pollution on health along with the current study results suggests that air pollution not only directly influences physical and mental health but also may negatively influence positive development, which is important for health and health-related development (e.g., developing healthy habits or reducing risky behavior). Early preventive strategies should be considered, as life stages are linked. One possible strategy is to reduce exposure and increase cooperation between agencies. The EPA can share hourly concentration levels with schools, for example, and schools can adjust classes accordingly, such as suspending outdoor programs during times with high air pollution concentration levels. Additionally, the EPA can issue warnings to parents so that proper protective action can be taken, such as wearing surgical masks when outdoor activities are inevitable but the concentration of pollution is high.

## Conclusion

In conclusion, this study found that long-term exposure to the three major air pollutants (i.e., PM_2.5_, PM_10_, and SO_2_) is associated with a lower level of happiness during early adolescence. These effects remained even after adjusting for individual- as well as district-level confounding variables. Furthermore, sensitivity analyses (e.g., adjusting for district size or number of participants) did not impact the results. However, long-term exposure to these three air pollutants did not have significant effects on the change in happiness for these participants during early adolescence (i.e., 7th grade to 9th grade). These results, combined with results from previous studies, indicate that air pollution, particularly the three air pollutants examined in this study, has detrimental effects on both adolescents’ positive and negative emotions.

### Additional file

Additional file 1: **Table S1.** Survey items used in the study (DOCX 26 kb)

## Data Availability

The datasets generated and analyzed during the current study are available at the Survey Research Data Archive (https://srda.sinica.edu.tw/). The governmental index used for district level covariates are publicly available, via: http://statdb.dbas.gov.taipei/pxweb2007-tp/dialog/statfile9.asp (Taipei city) http://pxweb.bas.ntpc.gov.tw/pxweb/dialog/statfile9_n.asp (Taipei county) https://civil.e-land.gov.tw/cp.aspx?n=B6700A96423BF15A (Yi-Lan county) (Last accessed April 17, 2019).

## Appendix

**Table 4:** Size of districts and number of subjects in the district^12^

Taipei city					
District zip code	Subjects	Siz(km^2^)	District zip code	Subjects	Siz(km^2^)
100	71	7.61	110	53	11.21
103	30	5.68	111	9	62.37
104	85	13.68	112	105	56.82
105	57	9.28	114	77	31.58
106	53	11.36	115	102	21.84
108	140	8.85	116	78	31.51
Taipei county					
207	19	63.38	236	77	29.58
208	49	49.21	238	99	33.13
220	165	23.14	241	155	16.32
221	21	71.24	242	78	19.74
222	22	20.58	243	3	19.16
223	34	144.35	247	6	7.43
226	3	71.34	248	4	34.86
231	37	120.23	249	66	39.49
234	116	5.71	251	73	70.65
235	115	20.14			
Yilan county					
260	103	29.41	266	13	144.22
262	63	101.43	268	83	38.87
263	73	38.48	269	95	79.86
264	14	111.91	270	63	89.02
265	62	11.34			

^1^Mean district size: 44.40

^2^Mean number of subjects per district: 64.28

## Abbreviations

AQI: Air Quality Index
EPA: Environmental Protection Administration
LUR: Land Use Regression
TYP: Taiwan Youth Project
WHO: World Health Organization
