# Supplementary material for: “Happiness in the air?” the effects of air pollution on adolescent happiness
Source: BMC Public Health. 2019 Jun 21;19:795. doi: 10.1186/s12889-019-7119-0 (PMC6588912; doi:10.1186/s12889-019-7119-0)
Supplement: Supplementary file 1 — Table S1. Survey items used in the study (DOCX 26 kb) [file 12889_2019_7119_MOESM1_ESM.docx]

Table S1 Survey items used in the present study

| Survey item (wave) | Response categories |
| --- | --- |
| In general, are you happy lately? (W1 to W3) | (1) Very happy  (2) Happy  (3) Unhappy  (4) Very unhappy |
| Gender (W1) | (0) Female(1) Male |
| Are you currently living with your parents? (W1) | (1) Living with biological parents  (2) Living with father and stepmother  (3) Living with mother and stepfather  (4) Living with father  (5) Living with mother  (6) Living with others: _______ |
| Average monthly family income(W1) | ________NT dollars |
| What was your GPA ranking in your class last semester? (W1) | (1) Exemplary (Top 5)  (2) Above average (Ranked 6th to 15^th^)  (3) Average (Ranked 16th-25^th^)  (4) Below average  (5) Barely passing |
| In the past year, did any of the following happen? (W1)^1^  (1) I severed ties with a close friend  (2) My sibling(s) ran away from home (Check and skip here if you are an only child)  (3) My parents divorced or separated  (4) My grades and ranking in class dropped  (5) I started wearing glasses or dental braces  (6) My family’s financial condition gets worse  (7) My parents argued more often  (8) My father/mother was absent from home more often  (9) I had conflicts with classmate(s)  (10) I got seriously ill or injured  (11) I transferred to another school  (12) I drank alcohol, smoked, or used drugs  (13) I caused some problems at school  (14) I was prohibited from participating in major school activities (e.g. sports, bands, or student clubs)  (15) I ran away from home  (16) My father/mother got laid off  (17) I argued with my parents more often  (18) My pet died  (19) My father/mother passed away  (20) A close relative of mine passed away | (0) No  (1) Yes  (2) Skip Question (Only child) |
| How much do the following descriptions of family life reflect your own family? (W1)  (1) My family discusses with each other before making a decision.  (2) My family likes to spend their free time together.  (3) Every family member participates in family activities.  (4) My family accepts each other's friends.  (5) I can get comfort from my family when I experience setbacks in life.  (6) I can rely on my family when I need help or advice. | (1) Very much  (2) Somewhat  (3) Not really  (4) Not at all |
| How much do you agree with the following descriptions of yourself? (W1)  (1) I cannot solve some personal problems.*  (2) I cannot control what happens to me.*  (3) I am optimistic about myself.  (4) I am satisfied with myself.  (5) Sometimes I feel useless.*  (6) Sometimes I feel that I don't have any desirable qualities.* | (1) Strongly Agree  (2) Agree  (3) Disagree  (4) Strongly disagree |
| In the past week, did you experience any of the following symptoms? If so, how severe were they?(W1)  (1) Loneliness  (2) Depression  (3) Excessive worry | (0) No  (1) Mild  (2) Moderate  (3) Serious  (4) Very serious |
| In the past year, have you ever done the following? Please answer based on the frequency.(W1)^2^  (1) Run away from home  (2) Skip class/school  (3) Vandalize  (4) Steal  (5) Engage in sexual behaviors  (6) Hit and injure someone  (7) Money bully someone  (8) Smoke or drink alcohol  (9) Chew betel nut  (10) Use drugs (e.g. pentazocine, amphetamine) or sniff glue | (1) Never  (2) Seldom (once or twice)  (3) Sometimes  (4) Often  (5) Always |
| How much do you agree with the following descriptions of your best friends?(W1)  (1) He/she/they care(s) about me  (2) He/she/they often help(s) me when I am in need  (3) He/she/they always comfort(s) me when I experience setbacks | (1) Never  (2) Seldom (once or twice)  (3) Sometimes  (4) Often  (5) Always |
| Do you think people in your neighborhood are willing to help others or do they only care about themselves? | (1) They are willing to help others  (2) They mind their own business  (3) About half and half |

*These items were revers coded

^1^Skip was coded as “no”

^2^Because very skewed distribution with original measure, each item was dichotomized into 1 (seldom to always) and 0 (never).
